# Selection and preliminary evaluation of superior individual plant in *Camellia oleifera*

**DOI:** 10.7717/peerj.20283

**Published:** 2025-11-20

**Authors:** Wenpei Song, Siqi Huang, Fang Li, Panfeng Tu, Yongquan Li, Bipei Zhang, Yi Wang, Jinghan Dou, Caiqin Li

**Affiliations:** 1 College of Horticulture and Landscape Architecture, Zhongkai University of Agriculture and Engineering, Guangzhou, Guangdong, China; 2 School of Mechanic and Electronic Engineering, Zhongkai University of Agriculture and Engineering, Guangzhou, Guangdong, China

**Keywords:** *Camellia oleifera*, Germplasm screening, Trait evaluation, Low-yield forest

## Abstract

*Camellia oleifera* exhibits significant economic and ecological value as a woody oil crop. However, widespread low-yield stands persist due to suboptimal agricultural management and historical neglect of scientific cultivation practices. This study conducted a systematic characterization of 25 elite *C. oleifera* germplasms with high fruit set but phenotypic variability in fruit morphology and yield components. Multi-dimensional assessments were performed at late-stage fruit development, focusing on architecture traits (height, canopy area), fruit morphological parameters (size, weight, pericarp thickness), and key economic indices including yield potential and oil content. Multivariate analysis revealed H2 as the top-performing genotype, demonstrating superior performance across all evaluated traits. Genotypes H16, H5 and H6 ranked second tier but require optimized agronomic practices to maximize yield potential. Hierarchical cluster analysis based on 17 quantitative traits classified the elite trees into five distinct phenotypic groups. These findings provide a scientific framework for genotype selection in low-yield forest restoration programs. The identified superior trees offer potential for regional production enhancement, while the established trait correlation inform targeted breeding strategies.

## Introduction

*Camellia oleifera* Abel. (commonly termed tea oil tree or tea seed tree) is an evergreen shrub of the genus *Camellia* in the family Theaceae. As one of the four globally significant woody oil crops alongside *Olea europaea* L., *Elaeis guineensis* Jacq., and *Cocos nucifera* L. (Ruilin, 2008), its seed oil contains abundant monounsaturated fatty acids and bioactive metabolites. These compounds exhibit anticancer, antioxidant, and immunomodulatory properties (Suealek et al., 2021), establishing it as a premium edible oil in international markets (Tan et al., 2020; Li et al., 2022).

China has cultivated *C. oleifera* for over 2,300 years (National Forestry and Grassland Administration, 2020) and harbors >90% of the 200+ identified *Camellia* species world wide. The oil, commonly termed ‘liquid gold’ or ‘Oriental olive oil’ (Yunai et al., 2008), exhibits high nutritional value, favorable organoleptic properties, and significant industrial processing potential (Song, Hendrich & Murphy, 1999; Nakpathom et al., 2017). Its predominant unsaturated fatty acids (80% oleic/linoleic acids) contribute to reduced blood pressure, lowered cholesterol levels, and attenuated aging markers (Liu et al., 2022).

Industrial development of *C. oleifera* in China has accelerated post-agricultural restructuring. Guangdong Province transitioned from production decline (1970s-1980s) to sustained expansion (Ruchun & Xiaomei, 2005), with cultivation area reaching 349,000 ha (2020) (Forestry Administration of Guangdong Province, 2021) coinciding with national seed production of 3.94 million metric tons (2021) (National Bureau of Statistics of China, 2021). Current government policies designate it as a strategic priority industry at the provincial level.

Breeding initiatives commencing in the 1960s enabled the selection of strains with stable yield and superior traits by the Chinese Academy of Agriculture and Forestry. A four-phase screening protocol (pre-selection, primary selection, re-selection, and superiority confirmation) was standardized in 1980 (Ruilin, 2010). Subsequent biotechnological advances (*e.g.*, molecular markers, tissue culture, transgenic techniques) had accelerated varietal improvement (Zhao, Yujuan & Wanchu, 2013). Given that economic trait assessment underpins the cultivation of economically viable forestry species (Yang et al., 2022), 25 *C. oleifera* accessions were preliminary evaluated in this study. Correlation analysis and principal component analysis (PCA) of tree architecture, fruit morphology, and economic traits were employed to identify high-yield *C. oleifera* accessions with superior fruit characteristics and establish a theoretical framework for low-yield forest transformation.

## Materials and Methods

### Experimental site description

The study was conducted in a *C. oleifera* plantation located in Heyuan City, Guangdong Province, China (23°N latitude, 115°E longtitude). Situated at the confluence of the Nanpan and Beipan Rivers—major tributaries of the Pearl River’s upper reaches—the site lies within the northeastern Guangdong-Hong Kong-Macao Greater Bay Area ecological zone. The region is characterized by a southern subtropical monsoon climate, with a mean annual temperature of 20.7 °C and mean annual precipitation ranging from 1,567 mm to 2,142.6 mm. Precipitation exhibits strong seasonal (April–June peak) and interannual variability, with a west-to-east rainfall gradient.

### Plant material selection

Twenty-five elite *C. oleifera* trees (designated H1–H25) were selected from a 30-year-old plantation established in the early 1990s. The experimental population met the following criteria: (i) seedling-origin stands with uniform age structure; (ii) absence of arthropogenic interference except annual harvesting; (iii) no significant incidence of pests/diseases during the study period (2020–2022); and (iv) superior growth performance confirmed through triennial monitoring of agronomic traits. Selected trees exhibited complete canopy architecture, symmetrical crown distribution, and consistent fruit-bearing capacity.

### Trait measurement methodologies

#### Tree growth indicators analysis

Plant height was measured using a digital hypsometer. Crown dimensions were recorded along east–west (C_ew_) and north-south (C_ns_) axes with a steel tape. Crown area (CA) was calculated using the ellipse formula (1):

(1) \begin{eqnarray*}CA= \frac{\pi }{4} \times \mathrm{Cew}\times {C}_{ns}.\end{eqnarray*}

#### Fruit characteristics analysis

At physiological muturity, fruits from each experimental unit were harvested. The fresh weight of individual plants was measured using a digital balance. The yield per square meter of canopy (Y_m_) was calculated according to Eq. (2):

(2) \begin{eqnarray*}{Y}_{\mathrm{m}}= \frac{Yp}{CA} \end{eqnarray*}

where Y_p_ represents annual yield per plant (kg tree^−1^).

Twenty-five fruits per plant were randomly sampled from four cardinal directions of the canopy peripheries. Fresh fruit weight (FFW) and fresh seed weight (FSW) were determined using an analytical balance. Fruit dimensions included transverse diameter (FTD) and longitudinal diameter (FLD), measured wit digital vernier calipers. Pericarp thickness was assessed at three equidistant points. The total number of fruits and seeds was also counted and recorded. Post-harvest parameters were calculated as Eqs. (3), (4), (5), and (6):

(3) \begin{eqnarray*}\text{Fruit shape index}= \frac{FLD}{FTD} \end{eqnarray*}

(4) \begin{eqnarray*}\text{Fresh seed rate}~(\%)= \frac{FSW}{FFW} \times 100\end{eqnarray*}

(5) \begin{eqnarray*}\text{Seed moisture content}~(\%)= \frac{FSW-DSW}{FSW} \times 100\end{eqnarray*}

(6) \begin{eqnarray*}\text{Dry seed rate}~(\%)= \frac{DSW}{FSW} \times 100\end{eqnarray*}

where *DSW* denotes dry seed weight after oven-drying at 65 °C for 48 h.

Seed oil content was quantified using Soxhlet extraction. Dried seed samples (2.00 g) were pulverized and placed in cellulose thimbles. Extraction was performed with petroleum ether (40–60 °C boiling range) for 7 h under reflux conditions. Post-extraction, thimbles were dried at 105 °C for 1 h, and reweighed. Seed oil content (SOC) was calculated as Eq. (7).

(7) \begin{eqnarray*}\mathrm{SOC}~(\%)= \frac{{W}_{0}-{\mathrm{W}}_{1}}{{\mathrm{W}}_{\mathrm{S}}} \times 100\end{eqnarray*}

where *W*_0_ is pre-extraction thimble weight, W_1_ represents post-extraction weight, and W_s_ represents sample weight.

### Statistical analysis

All data are presented as the mean ± standard error of the mean (SEM). Statistical analyses were conducted using SPSS Statistics software (version 13.0; IBM Corp., Armonk, NY, USA) and WPS Office (Kingsoft Crop., Zhuhai, China). For comparative analysis between samples, one-way analysis of variance (ANOVA) followed by Duncan’s multiple range test was employed to determine statistically significant differences among experimental samples. A significance threshold of *p* < 0.05 was applied for all comparisons.

Multivariate statistical analyses, including principal component analysis (PCA) and Pearson’s correlation analysis, were performed using OriginPro software (version 2021; OriginLab Corp., Northampton, MA, USA). PCA was utilized for dimensionality reduction and pattern recognition in miltidimensional datasets, while Pearson’s correlation analysis quantified linear relationships between variables. Results visualized through correlation heatmap and hierarchical cluster diagram were generated using the integrated tools in OriginPro.

## Results

### Phenotypic variations analysis of elite *C. oleifera* trees

#### Tree architecture traits

Phenotypic variation was quantified using coefficient of variation (CV) across 20 morphological parameters in 25 preliminarily selected elite trees (Table 1). Four key growth traits exhibited significant variation (CV > 14%). Tree height showed the lowest variation (CV = 14.27%) with mean 2.44 m, ranging from 1.80 m (H18) to 3.10 m (H22). In contrast, canopy area exhibited remarkable phenotypic diversity (CV = 44.67%), averaging 4.01 m^2^, with extremes observed in H6 (7.19 m^2^) and H10 (1.39 m^2^) (Table S1).

**Table 1:** Phenotypic variation of 19 quantitative traits in 25 elite *C. oleifera* genotypes.

Terms	Traits	Average	Standard deviation	Maximum	Minimum	Variation coefficient (%)
Tree architecture	Plant height (m)	2.44	0.35	3.10	1.80	14.27
	Crown width (east–west) (m)	2.19	0.53	3.15	1.35	24.09
	Crown width (north–south) (m)	2.23	0.55	3.30	1.20	24.68
	Canopy area (m^2^)	4.01	1.81	7.19	1.39	44.67
Fruit yield	Yield per plant (kg)	1.58	1.26	5.67	0.33	77.73
	Yield per square meter of canopy (kg/m^2^)	0.46	0.35	1.76	0.11	81.88
	land equivalent oil yield (kg/ha)	360.39	276.46	1,380.66	82.55	81.90
Fruit quality	Fruit weight (g)	31.14	8.47	50.00	17.22	34.81
	Pericarp weight (g)	17.55	5.33	32.56	8.17	34.83
	Pericarp thickness (mm)	4.21	0.75	6.57	2.78	20.94
	Fruit longitudinal diameter (mm)	38.60	2.76	44.56	33.47	10.87
	Fruit transverse diameter (mm)	38.53	3.96	47.65	31.40	13.93
	Fruit shape index	1.01	0.07	1.19	0.86	11.04
Seed and Oil	Seed number per plant (seed)	4.50	1.19	7.00	2.00	43.72
	100-seed weight (g)	329.37	75.81	509.73	220.44	37.30
	Seed moisture content (%)	44.19	11.71	87.28	28.51	30.47
	Fresh seed rate (%)	43.90	5.26	56.65	31.86	16.85
	Dry seed rate (%)	58.69	7.38	71.49	41.00	15.57
	Seed oil content (%)	36.14	5.60	45.84	23.17	15.95

#### Fruit yield traits

Notably, three yield-related parameters, yield per plant (77.73%), yield per square meterof canopy (81.88%), and land equivalent oil yield (81.90%), exhibited exceptionally high variation (Table 1), suggesting strong potential for clonal selection. Genotypic performance analysis revealed striking productivity disparities: H16 showed superior yield capacity (3.31 kg/plant), representing a 9.7-fold advantage over H15 (0.34 kg/plant; Table S2). Spatial yield assessment showed mean values of 0.46 kg/m^2^ for yield per square meter of canopy and 337.57 kg/ha for land equivalent oil yield, with extreme genotypes H16 (1.76 kg/m^2^; 1,380.66 kg/ha) and H7 (0.11 kg/m^2^; 82.55 kg/ha) defining the phenotypic spectrum.

#### Fruit quality traits

**Figure 1:**
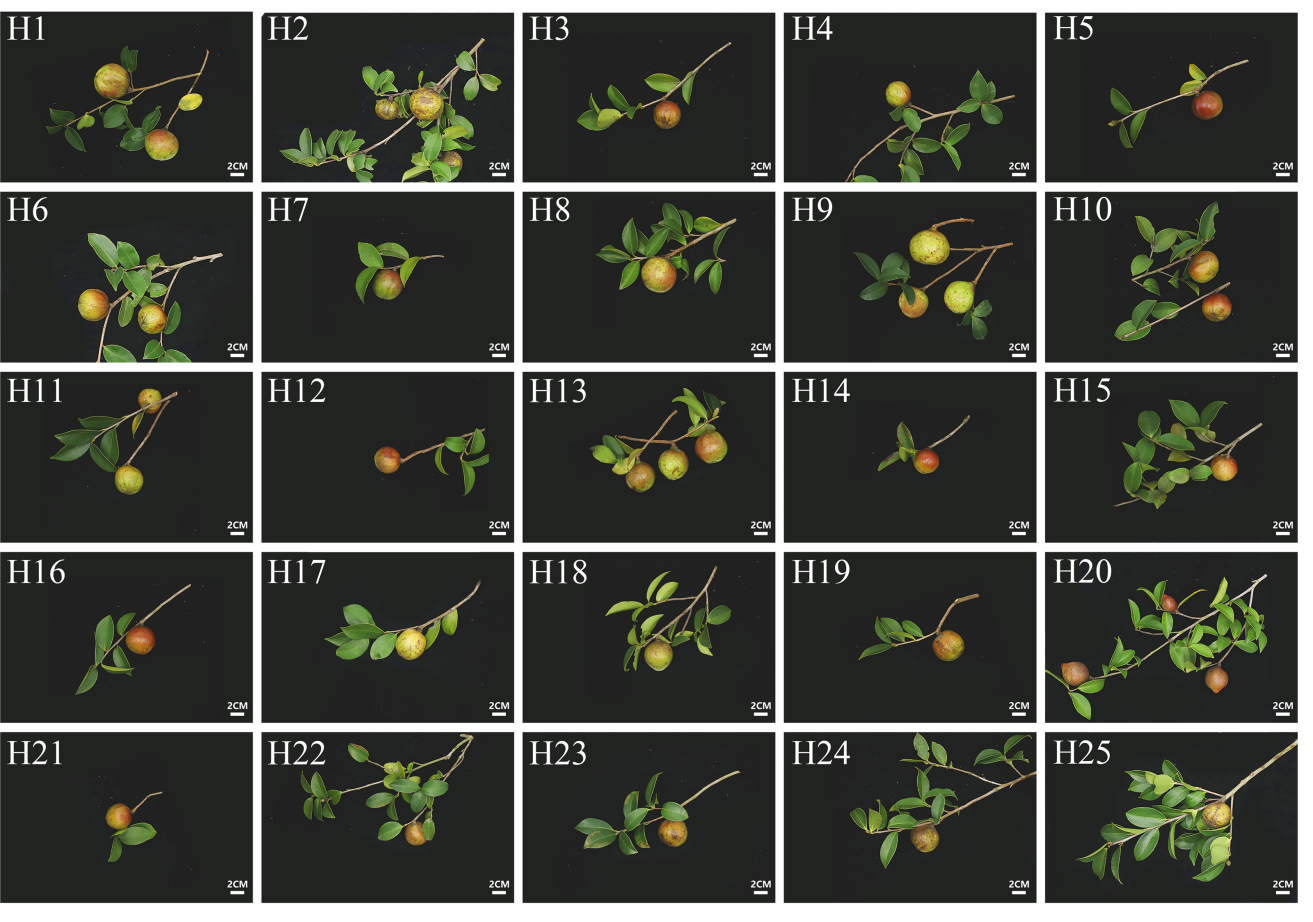
Fruit morpholoty of 25 elite *C. oleifera.* genotypes. Bar = two cm.

Six quality-related parameters revealed genotype-specific variation patterns. Three morphological traits showed relatively stable performance (Table 1): fruit longitudinal diameter (CV = 10.87%), fruit shape index (CV = 11.04%), and fruit transverse diameter (CV = 13.93%). Fruit shape analysis based on fruit shape index (FSI) values revealed predominantly globose fruits (56%; 0.9 < FSI < 1), followed by oblate fruits (40%; FSI > 1), with only 4% showing ovate morphology (0.8 < FSI < 0.9; Fig. 1, Table S3). Single fruit weight and pericarp weight exhibited substantial variability (CV = 34.81% and 34.83%, respectively), with H2, H7, H9, and H16 exceeding 40 g, while H23 showed the smallest fruit size (17.22 g). Pericarp weight ranged from 32.56 g (H7) to 8.17 g (H20), with a mean value of 17.55 g (Table 1, Table S3).

#### Seed and oil characteristic

Six seed-related parameters revealed distinct variation patterns. Dry seed rate (CV = 15.57%) and seed oil content (CV = 15.95%) showed moderate phenotypic stability (Table 1). Dry seed rate ranged from 71.49% (H20) to 41.00% (H1), with a mean value of 58.29% (Table S4). Seed oil content averaged 36.14%, with H24 exhibiting the highest value (45.84%) and H1 the lowest (23.17%). Seed number per plant showed remarkable variability (CV = 43.72%), range from 7 seeds (H16) to 2 seeds (H23).

### Correlation analysis and interpretation

**Figure 2:**
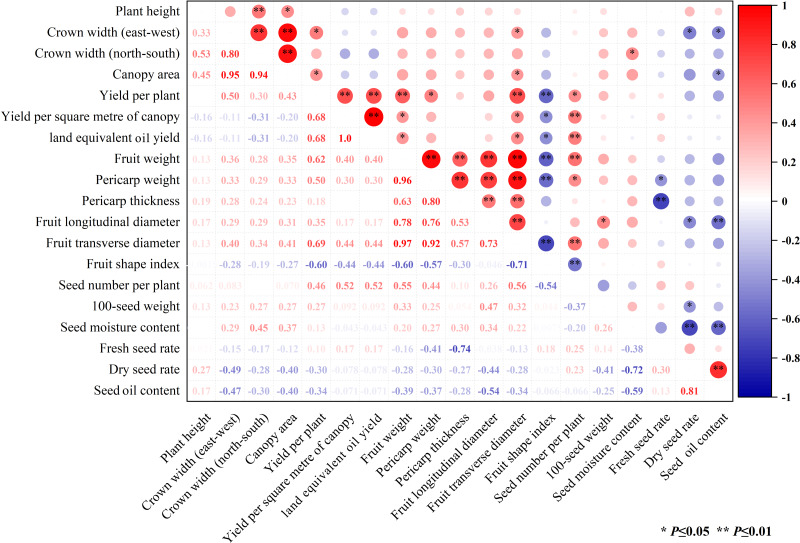
Cluster correlation heat map among various quantitative traits.

A Pearson correlation analysis was conducted on 19 individual indicators, as depicted in Fig. 2. The results revealed that 33 pairs of indicators exhibited an extremely significant correlation (*P* < 0.01), while 21 pairs demonstrated a significant correlation (*P* < 0.05). Focusing on the comprehensive trait indicators, fruit weight displayed highly robust positive correlations with several key morphological and yield-related traits. Specifically, it showed extremely significant positive correlations with fruit transverse diameter (*r* = 0.97), pericarp weight (*r* = 0.96), fruit longitudinal diameter (*r* = 0.78), pericarp thickness (*r* = 0.63), yield per plant (*r* = 0.62), and seed number per plant (*r* = 0.55). Conversely, fruit weight had an extremely significantly negative correlation with the fruit shape index (*r* = −0.60), indicating an inverse relationship between these two traits. Regarding fruit pericarp characteristics, pericarp thickness was extremely significant positively correlated with pericarp weight (*r* = 0.80), suggesting that thicker pericarp were generally associated with heavier pericarps. Additionally, pericarp thickness showed a significant negative correlation with the fresh seed rate (*r* = −0.74), implying that as pericarp thicker increases, the proportion of fresh seeds tends to decrease. In the context of seed quality and yield, seed oil content exhibited an extremely significant positive correlation with dry seed yield (*r* = 0.81), indicating that higher dry seed yields were often accompanied by increased seed oil content. On the other hand, seed oil content was extremely significantly negatively correlated with seed moisture content (*r* = −0.59) and fruit longitudinal diameter (−0.54), suggesting that lower seed moisture content and smaller fruit longitudinal diameters may contribute to higher seed oil content. Notably, as expected, the three yield-related parameters (yield per plant, yield per square meter of canopy, and land equivalent oil yield) demonstrated a highly significant positive correlation among themselves, which was consistent with the fundamental principles of crop yield assessment and highlights the interconnectedness of these yield-determining factors.

### Preliminary traits evaluation *via* PCA

The PCA method was used to preliminary evaluate 11 morphological and productive traits across 25 elite *C. oleifera* genotypes. The cumulative variance contribution of the first five principal components (PCs) reached 85.25% (Table 2), indicating these components sufficiently captured the multidimensional trait variability. Detailed loadings and variance contributions were provided in Table S5. PC1 accounted for the largest proportion of variance (36.06%) and was primarily associated with fruit size traits, including fruit weight, fruit transverse diameter, and pericarp weight. PC2 explained 19.92% of the variance, with major contributions from yield per square metre of canopy, land equivalent oil yield, and seed number per plant, reflecting yield-related performance. PC3 (11.69% variance) was strongly correlated with plant height, dry seed rate, and seed oil content, representing tree vigor and seed quality attributes. PC4 (10.42% variance) combined fresh seed yield, yield per plant, pericarp thickness, and pericarp weight, indicating pericarp-related yield components. PC5 (7.16% variance) was predominantly influenced by fruit longitudinal diameter and 100-seed weight.

**Table 2:** Analysis of variance for PCA.

Principal component	Eigenvalue	Contribution (%)	Cumulative contribution (%)
PC1	6.85	36.06	36.06
PC2	3.78	19.92	55.98
PC3	2.22	11.69	67.67
PC4	1.98	10.42	78.09
PC5	1.36	7.16	85.25

**Figure 3:**
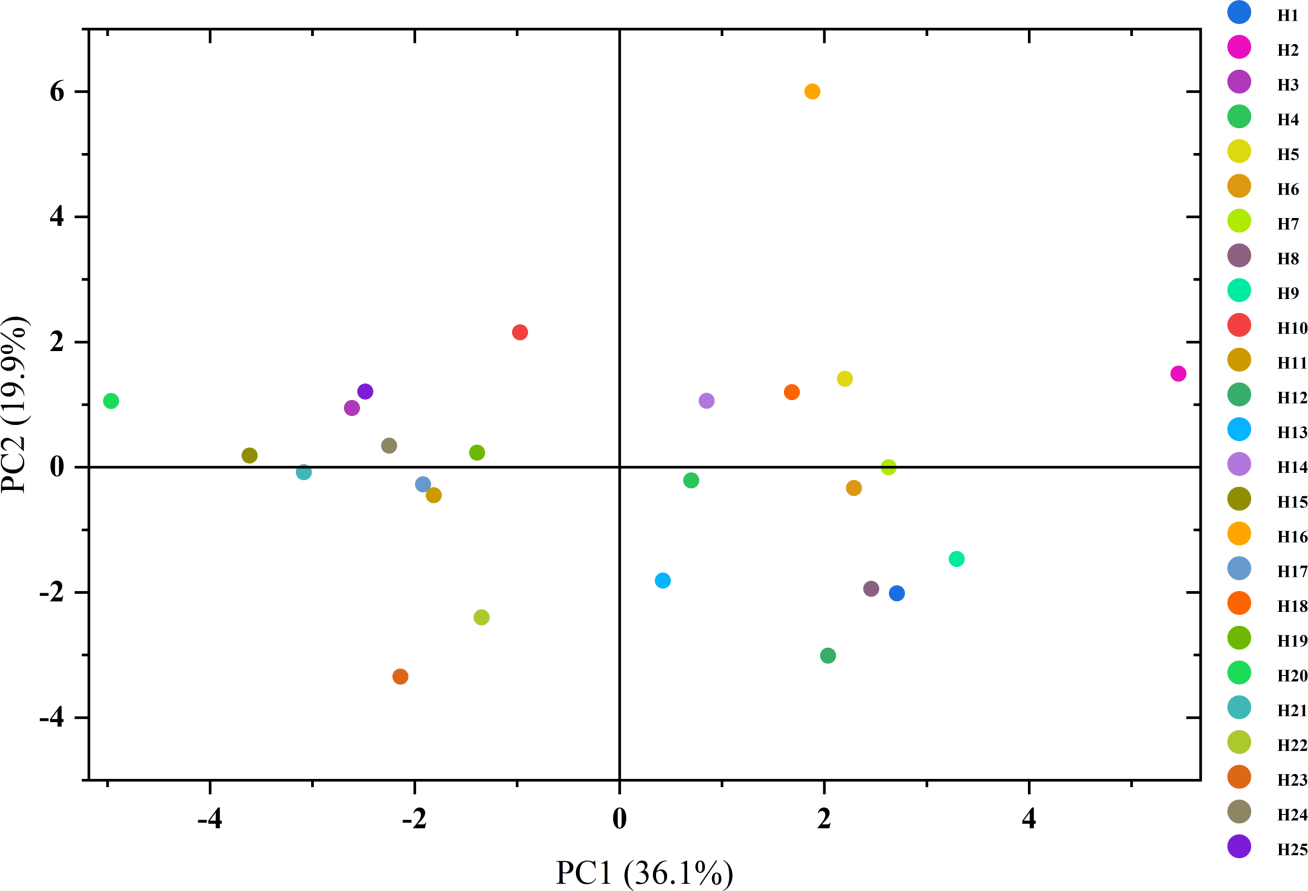
Two-dimensional principal component analysis of quantitative traits in 25 elite *C. oleifera* genotypes. Spatial distribution of genotypes visualized in the PC1-PC2 plane derivedfrom PCA. The proportion in parenthese is the variance of the various components. Each data point represents an elite tree. PC, principal component.

A biplot of PC1 *vs.* PC2 (Fig. 3) visualized the phenotypic differentiation among genotypes. To synthesize multi-trait performance, a composite index was calculated by weighting each PC with its proportion of explained variance. The resulting scores (Table 3) revealed genotype H2 as the top performer (1.31), followed by H16 (1.03), H5 (0.55), and H6 (0.50). Conversely, genotypes H15, H21, and H23 exhibited the lowest composite scores (<−0.50), indicating inferior overall performance.

**Table 3:** Eigenvalue of each principal component.

Genotypes	*F1*	*F2*	*F3*	*F4*	*F5*	*F*	Rank
H1	1.03	−1.03	−1.92	0.12	−0.32	−0.08	13
H2	2.08	0.77	0.52	1.31	0.40	1.31	1
H3	−1.00	0.49	−0.14	−0.41	−0.49	−0.42	22
H4	0.27	−0.11	1.46	0.26	0.16	0.34	6
H5	0.84	0.73	0.99	−0.60	−0.50	0.55	3
H6	0.87	−0.17	1.25	0.56	−0.88	0.50	4
H7	1.00	0.00	0.61	−3.71	1.67	0.20	9
H8	0.94	−1.00	0.31	0.50	0.00	0.27	8
H9	1.26	−0.75	0.34	0.23	−0.41	0.40	5
H10	−0.37	1.11	−1.42	−0.14	0.23	−0.10	14
H11	−0.69	−0.23	−0.30	−0.33	0.58	−0.38	20
H12	0.78	−1.55	−0.21	−0.66	−0.48	−0.18	15
H13	0.16	−0.93	−2.28	0.58	2.59	−0.19	16
H14	0.32	0.55	0.30	0.38	−0.74	0.29	7
H15	−1.38	0.10	0.14	−0.06	−0.69	−0.60	23
H16	0.72	3.09	−0.28	0.36	0.14	1.03	2
H17	−0.73	−0.14	−0.24	0.33	0.21	−0.32	18
H18	0.64	0.62	−1.85	0.60	−1.51	0.10	10
H19	−0.53	0.12	0.97	−0.29	0.52	−0.05	12
H20	−1.90	0.54	0.35	1.04	1.23	−0.40	21
H21	−1.18	−0.04	−0.21	−1.00	−1.82	−0.80	24
H22	−0.51	−1.23	1.56	1.78	1.08	0.02	11
H23	−0.82	−1.72	−0.21	−0.26	−1.49	−0.92	25
H24	−0.86	0.18	0.37	−0.16	0.55	−0.24	17
H25	−0.95	0.62	−0.11	−0.44	−0.05	−0.33	19

**Notes.**

*F*, Comprehensive score; *F1*–*F5*, Eigenvalue of PC1 (principal component 1) to PC5.

### Hierarchical cluster analysis of traits

**Figure 4:**
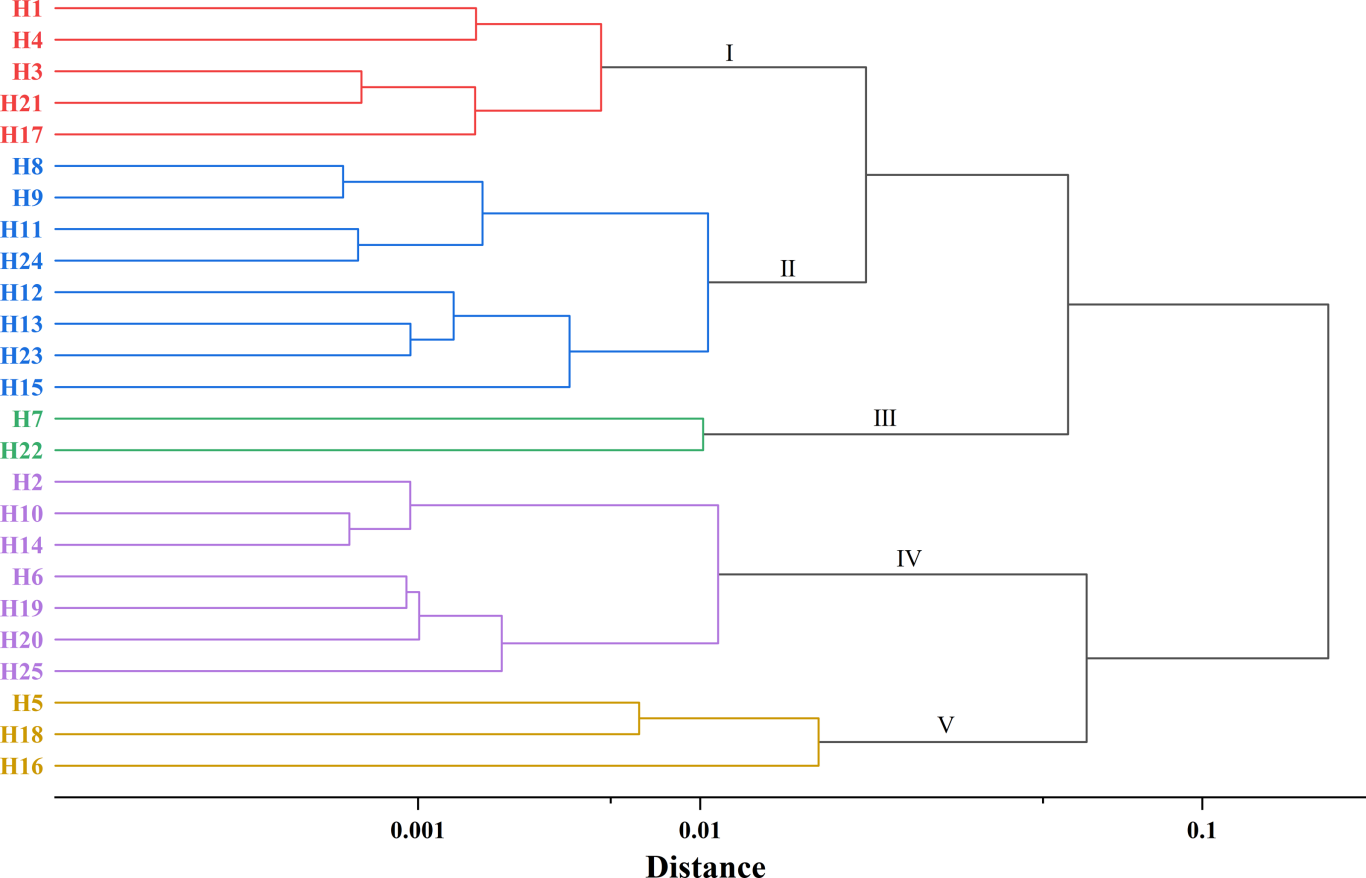
Hierarchical cluster diagram of 25 elite *C. oleifera* genotypes.

The initial cohort of 25 elite trees was categorized into five distinct groups based on phenotypic and agronomic traits (Fig. 4, Table S6). Group I comprised five trees (H1, H3, H4, H17, and H21), representing 20% of the total assessed germplasm. This group was characterized by small fruit size (mean fruit weight: 28.41 g), low productivity (mean land equivalent oil yield: 247.92 kg/ha), and reduced seed oil content (34.28%). Group II contained eight superior trees (H8, H9, H11, H12, H13, H15, H23, and H24), accounting for 32% of the population. Key traits included small fruits (29.77 g), thick pericarp (4.30 cm), and low oil yield (184.92 kg/ha) with reduced dry seed rate (54.96%) and seed oil content (34.85%). However, this group exhibited significantly higher 100-seed weight (354.79 g) and seed moisture content (50.14%) compared to other group. Group III included two superior trees (H7 and H22), representing 8% of the population. This group demonstrated the lowest oil productivity (97.675 kg/ha) and seed moisture content (35.96%). Notably, it showed superior 100-seed weight (388.50 g), dry seed rate (64.08%), and seed oil content (39.24%). Group IV contained seven elite trees (H2, H6, H10, H14, H19, H20, and H25), comprising 28% of the population. This high-performing group exhibited superior oil yield (413.67 kg/ha), fresh seed rate (47.00%), dry seed rate (61.62%), and seed oil content (37.13%). These genotypes were characterized by thin pericarp (3.87 cm) and moderate seed moisture content (38.39%). Group V included three elite trees (H5, H16, and H18), representing 12% of the population. This group was distinguished by large fruit size (37.21 g), highest oil productivity (876.41 kg/ha), and superior long fruit transverse diameter (41.62 mm) and thick pericarp (4.49 cm). Notably, these genotypes exhibited the lowest 100-seed weight (296.36 g) among all groups.

## Discussion

In 1977, China *C. oleifera* Seed Selection and Breeding Collaborative Group established the “Criteria and Methods for Selecting Excellent *C. oleifera* Trees”, which stipulated: (1) minimum tree age of 15 years; (2) desirable tree architecture with minimal pest/disease incidence; (3), average annual fruit yield exceeding 1 kg/m^2^ (≥0.5 kg) with uniform fruit size; and (4) fresh seed yield ≥40%, dry seed yield ≥ 25%, and seed oil content ≥42% (Shujie et al., 1980). Based on established selection criteria, we conducted a preliminary phenotypic evaluation of 25 *C. oleifera* monocultures, focus on the tree architecture, fruit characteristics, and economic traits. Through a combination of correlation analysis, principal component analysis (PCA), and multi-trait preliminary screening, four superior genotypes (H2, H16, H5, and H6) were identified as having optimal fruit quality parameters. Among these selected genotypes, genotype H2 demonstrated superior performance in fresh fruit weight (50.00 g) and fresh seed yield (47.12%) compared to other cultivars. However, this genotype exhibited a significantly higher seed moisture content (43.80%) coupled with relatively lower seed oil content. These findings suggested that while H2 showed promise for commercial production, optimized harvest timing protocols should be be implemented to mitigate potential quality deterioration associated with premature harvesting. It should be noted that these four genotypes represent preliminary screening results requiring validation through multi-year replicated trials. Notably, genotypes H11, H15, H19, and H24 exhibited superior seed oil contents (>40%) but failed to meet yield thresholds, indicating potential for improvement through targeted horticultural interventions. Horticultural management strategies may be optimized based on monoculture types to enhance yield, including weed control and soil aeration, balanced fertilization regimes, pruning practices, soil amendment, and integrated pest management for new forests; intercropping and non-target species removal for mixed forests; and forest thinning, large seedling replacement, and species renewal for aging forests (Yongyou, 2020).

With regard to the magnitude of variation in various traits of *C. oleifera*, the variability of tree traits, such as plant height and crown width was not significant, and the coefficients of variation of most fruit and economic traits were small and stable. Among them, the coefficients of variation of fresh fruit weight, seed number, and yield were large (34.81%, 43.72%, and 81.90%, respectively), indicating that *C. oleifera* maybe has a rich diversity related to fresh fruit weight, seed index, and yield index. The greater the magnitude of trait variation within a certain population, the more favorable are the chances for germplasm variation, genetic manipulation and superior resources (Lirong, Gengrui & Weichao, 2005). Thus, some *C. oleifera* traits may evolve more quickly, with high fruit weight, seed yield, and oil content, acting as important reference indicators in *C. oleifera* breeding. These findings were consistent with those of a previous study (Chun et al., 2011).

Complex correlations were observed among the various economic trait indices of 25 common *C. oleifera* plants. In this study, single fruit weight was highly significantly and positively correlated with pericarp thickness, number of seeds, total number of fruits, and single plant yield. Pericarp thickness was highly significantly and negatively correlated with fresh seed yield, indicating that the greater the pericarp thickness, the lower the fresh seed yield. The seed traits of oil trees are related to economic value, as reported by Shaofeng et al. (2007). This indicates that in *C. oleifera* cultivation, the selection of *C. oleifera* fruits with a high number of seeds, large and full seeds, and thin peels as breeding materials can improve seed oil content.

Cluster analysis was performed on 25 elite trees, which were clustered into five distinct groups (Group I to Group V). Among them, Group V demonstrated significantly larger fruit size and higher yield compared to other clusters. Group IV was distinguished by thinner pericarp thickness while maintaining high yield and oil content. These findings suggested that Groups IV and Groups V represented valuable elite germplasm resources. Specifically, Groups V could serve as an ideal parental line for breeding programs targeting large-fruited cultivars with high productivity, while Groups IV offers unique potential for developing thin-skinned varieties with optimal oil yield. The combination of these traits in hybrid progeny could facilitate the development of superior cultivars with multiple desirable characteristics.

## Conclusions

There is rich variation in various traits among 25 initially selected elite trees of *C. oleifera* in Heyuan. Among them, H2, H16, H5, and H6 showed better comprehensive performance and can be exploited and utilized as excellent germplasm resources to improve economic benefits. Fruit weight, pericarp thickness, fresh seed rate, dry seed rate, Seed oil content, and yield per plant can be used as the core indicators for the evaluation of elite trees of *C. oleifera* in Heyuan.

## Supplemental Information

10.7717/peerj.20283/supp-1 Supplemental Information 1 Raw data

10.7717/peerj.20283/supp-2 Supplemental Information 2 Supplementary tables

## Additional Information and Declarations

### Data Availability

The following information was supplied regarding data availability: The raw measurements are available in the Supplementary Files.
