# Supplementary material for: Selection and preliminary evaluation of superior individual plant in Camellia oleifera
Source: PeerJ. 2025 Nov 20;13:e20283. doi: 10.7717/peerj.20283 (PMC12640634; doi:10.7717/peerj.20283)
Supplement: Supplemental Information 2 [file peerj-13-20283-s002.docx]

Table S1 Comparative analysis of tree architecture traits

| Genotypes | Plant height  (m) | Crown width (east-west)  (m) | Crown width (north-south)  (m) | Canopy area  (m^2^) |
| --- | --- | --- | --- | --- |
| H1 | 2.15 | 2.50 | 2.50 | 4.91 |
| H2 | 2.50 | 2.85 | 2.88 | 6.45 |
| H3 | 2.03 | 1.55 | 1.83 | 2.23 |
| H4 | 3.00 | 2.57 | 2.65 | 5.35 |
| H5 | 2.83 | 2.55 | 2.25 | 4.51 |
| H6 | 2.45 | 3.05 | 3.00 | 7.19 |
| H7 | 2.75 | 1.90 | 2.18 | 3.25 |
| H8 | 2.75 | 2.90 | 2.90 | 6.61 |
| H9 | 2.35 | 3.15 | 2.90 | 7.17 |
| H10 | 1.94 | 1.48 | 1.20 | 1.39 |
| H11 | 2.52 | 1.90 | 1.85 | 2.76 |
| H12 | 2.85 | 2.15 | 3.30 | 5.57 |
| H13 | 2.25 | 2.00 | 1.90 | 2.98 |
| H14 | 2.15 | 2.45 | 2.35 | 4.52 |
| H15 | 2.15 | 2.00 | 1.60 | 2.51 |
| H16 | 2.55 | 1.60 | 1.50 | 1.88 |
| H17 | 2.25 | 1.95 | 2.20 | 3.37 |
| H18 | 1.80 | 2.40 | 1.88 | 3.54 |
| H19 | 2.75 | 2.18 | 2.20 | 3.77 |
| H20 | 2.60 | 1.35 | 1.70 | 1.80 |
| H21 | 1.95 | 1.75 | 2.00 | 2.75 |
| H22 | 3.10 | 2.70 | 3.10 | 6.57 |
| H23 | 2.65 | 2.75 | 2.40 | 5.18 |
| H24 | 2.21 | 1.76 | 2.24 | 3.10 |
| H25 | 2.42 | 1.53 | 1.60 | 1.92 |
| CV (%) | 14.27 | 24.09 | 24.68 | 44.67 |

Table S2 Comparative analysis of fruit yield traits

| Genotypes | Yield per plant  (kg) | Yield per square metre of canopy  (kg/m^2^) | land equivalent oil yield (kg/ha) |
| --- | --- | --- | --- |
| H1 | 2.26 | 0.46 | 361.32 |
| H2 | 5.67 | 0.88 | 691.06 |
| H3 | 0.57 | 0.26 | 200.63 |
| H4 | 1.60 | 0.30 | 234.68 |
| H5 | 2.97 | 0.66 | 517.73 |
| H6 | 3.18 | 0.44 | 347.66 |
| H7 | 0.34 | 0.11 | 82.55 |
| H8 | 1.69 | 0.26 | 201.34 |
| H9 | 1.75 | 0.24 | 191.33 |
| H10 | 0.95 | 0.68 | 532.21 |
| H11 | 0.87 | 0.32 | 247.98 |
| H12 | 1.15 | 0.21 | 162.37 |
| H13 | 0.88 | 0.30 | 232.54 |
| H14 | 2.37 | 0.52 | 411.36 |
| H15 | 0.33 | 0.13 | 102.24 |
| H16 | 3.31 | 1.76 | 1380.66 |
| H17 | 1.19 | 0.35 | 277.25 |
| H18 | 3.30 | 0.93 | 730.85 |
| H19 | 1.49 | 0.39 | 309.69 |
| H20 | 0.58 | 0.32 | 251.24 |
| H21 | 0.58 | 0.21 | 165.72 |
| H22 | 0.94 | 0.14 | 112.80 |
| H23 | 0.87 | 0.17 | 131.92 |
| H24 | 0.83 | 0.27 | 209.60 |
| H25 | 0.86 | 0.45 | 352.45 |
| CV (%) | 77.73 | 81.88 | 81.90 |

Table S3 Comparison of fruit quality traits

| Genotypes | Fruit weight  (g) | Pericarp weight  (g) | Pericarp thickness  (mm) | Fruit longitudinal diameter  (mm) | Fruit transverse diameter  (mm) | Fruit shape index |
| --- | --- | --- | --- | --- | --- | --- |
| H1 | 33.88±1.06c | 20.95±0.65cde | 4.82±0.14bc | 41.90±0.57bc | 41.20±0.41cd | 1.0179 |
| H2 | 50.00±2.03a | 26.37±1.13b | 4.41±0.16cde | 41.02±0.39cd | 47.65±0.78a | 0.8638 |
| H3 | 26.96±2.25de | 14.71±1.07g | 3.45±0.12k | 35.87±0.90jk | 36.30±1.18hijk | 0.9962 |
| H4 | 33.70±0.77c | 17.86±0.41f | 3.95±0.09fghij | 39.53±1.16defg | 40.19±0.48cdef | 0.9863 |
| H5 | 38.20±0.96bc | 22.06±0.51cd | 5.12±0.15b | 39.05±0.32defgh | 42.31±0.42cd | 0.9239 |
| H6 | 36.82±1.36bc | 20.69±1.05cdef | 4.19±0.13defg | 38.91±0.44defghi | 41.87±0.56cd | 0.9316 |
| H7 | 48.80±5.70a | 32.56±3.04a | 6.57±0.22a | 43.75±1.72ab | 45.00±2.04b | 0.9751 |
| H8 | 37.04±2.26bc | 19.90±1.03def | 4.54±0.13cd | 40.57±0.72cde | 40.88±0.95cde | 0.9980 |
| H9 | 41.29±1.94b | 22.96±0.82c | 4.40±0.13cde | 41.82±0.76bc | 42.86±0.81bc | 0.9800 |
| H10 | 33.18±1.17de | 17.82±1.07f | 3.70±0.11hijk | 38.17±0.52fghij | 40.20±0.71cdef | 0.9520 |
| H11 | 26.24±1.17de | 14.78±0.66g | 4.51±0.09cd | 36.63±0.50ij | 35.96±0.73hijk | 1.0261 |
| H12 | 33.22±1.87c | 20.65±0.97cdef | 4.83±0.12bc | 39.75±0.74cdefg | 40.03±0.72def | 0.9958 |
| H13 | 35.28±1.65c | 18.71±1.01ef | 4.23±0.12defg | 44.56±0.93a | 38.19±1.16efgh | 1.1888 |
| H14 | 33.93±1.51c | 17.95±0.76f | 4.13±0.14defgh | 38.60±0.57efghi | 40.40±0.81cde | 0.9588 |
| H15 | 21.81±1.19efg | 11.35±0.53h | 3.56±0.13ijk | 34.18±0.47kl | 34.46±0.79jk | 0.9965 |
| H16 | 40.63±1.15b | 21.94±0.61cd | 4.15±0.09defg | 39.49±0.38defg | 42.89±0.59bc | 0.9235 |
| H17 | 25.25±2.02de | 13.77±0.96gh | 3.84±0.09ghijk | 36.89±0.71hij | 35.11±1.11ijk | 1.0633 |
| H18 | 32.79±1.35c | 18.81±0.65ef | 4.20±0.14defg | 39.94±0.51cdef | 39.66±0.52defg | 1.0082 |
| H19 | 27.29±0.95d | 15.08±0.51g | 4.37±0.13def | 38.63±0.40efghi | 37.41±0.46ghi | 1.0338 |
| H20 | 19.06±1.34fg | 8.17±0.53j | 2.78±0.13l | 37.68±0.72fghij | 31.98±0.85l | 1.1847 |
| H21 | 22.28±2.36defg | 13.46±1.32gh | 3.97±0.11efghi | 33.47±1.11l | 33.85±1.37kl | 0.9982 |
| H22 | 23.56±0.91def | 11.82±0.45h | 2.86±0.08l | 37.42±0.54ghij | 36.38±0.57hijk | 1.0330 |
| H23 | 17.22±1.02g | 11.37±0.69h | 4.51±0.17cd | 34.39±0.51kl | 31.40±0.73l | 1.1043 |
| H24 | 26.06±1.57de | 13.94±0.86gh | 3.81±0.17ghijk | 38.41±0.77efghi | 37.58±0.76fghi | 1.0237 |
| H25 | 26.42±1.42de | 14.99±0.83g | 3.53±0.15jk | 36.58±0.72ij | 36.98±0.66hij | 0.9906 |
| CV (%) | 34.81 | 34.83 | 20.94 | 10.87 | 13.93 | 11.04 |

Table S4 Comparative Analysis of seed and oil characteristics

| Genotypes | Seed number per plant  (seed) | 100-seed weight  (g) | Seed moisture content  (%) | Fresh seed rate  (%) | Dry seed rate  (%) | Seed oil content  (%) |
| --- | --- | --- | --- | --- | --- | --- |
| H1 | 3±0.25ghijk | 404.59±22.31bcd | 59.00±1.48b | 37.96±1.18f | 41.00±1.46k | 23.17±1.15l |
| H2 | 5±0.36bcde | 500.80±23.05a | 43.80±0.91ef | 47.12±0.57bcd | 56.25±0.91gh | 32.47±1.51hij |
| H3 | 5±0.45cdefg | 278.59±12.97hijk | 36.24±2.55ijkl | 43.86±1.27cde | 63.74±2.54bcd | 37.43±1.33defg |
| H4 | 6±0.22ab | 276.74±8.81hijk | 38.64±0.99hijk | 46.88±0.46bcd | 61.34±0.97def | 34.77±1.01efghi |
| H5 | 6±0.36a | 266.65±11.99ijk | 38.24±0.63hijkl | 42.08±0.58e | 61.75±0.62def | 34.54±2.83fghi |
| H6 | 5±0.27bcd | 317.90±11.03efghi | 41.48±1.27fgh | 44.00±1.91cde | 58.55±1.26fg | 37.47±0.55defg |
| H7 | 5±0.74bcd | 311.90±38.60efghi | 34.14±1.61kl | 31.86±2.53g | 65.92±1.67bc | 38.15±1.38def |
| H8 | 5±0.44bcde | 351.61±12.09cdefg | 51.16±0.89d | 45.36±0.96cde | 48.87±0.91i | 28.29±0.74jk |
| H9 | 6±0.35ab | 324.82±11.00efghi | 50.44±0.91d | 43.32±1.37cde | 49.46±0.91i | 29.46±0.82jk |
| H10 | 4±0.24cdefgh | 359.81±20.19cdef | 43.19±2.07efg | 46.42±1.74bcd | 56.83±2.07gh | 35.00±0.62efghi |
| H11 | 3±0.21jkl | 408.71±15.35bc | 40.89±1.61fghi | 43.33±1.11cde | 59.04±1.60efg | 40.58±0.88bcd |
| H12 | 4±0.38efghij | 337.43±17.23defghi | 87.28±3.26a | 37.04±1.18f | 53.73±0.86h | 32.65±0.93ghij |
| H13 | 3±0.21hijk | 509.73±20.98a | 53.04±1.23cd | 47.16±1.21bcd | 46.89±1.23ij | 27.39±2.04k |
| H14 | 6±0.39ab | 283.97±13.56ghijk | 39.14±1.05ghij | 46.98±0.59bcd | 60.86±1.05def | 31.21±1.03ijk |
| H15 | 4±0.30fghij | 271.68±13.64hijk | 36.74±1.38ijkl | 47.45±1.23bc | 63.26±1.38bcd | 40.28±0.51bcd |
| H16 | 7±0.38a | 340.45±61.92cdefgh | 37.21±0.66hijkl | 45.85±0.72bcde | 62.79±0.66cdef | 39.54±0.96bcde |
| H17 | 3±0.31ijkl | 382.53±14.95cde | 38.73±0.87hijk | 44.54±0.87cde | 61.27±0.87def | 37.21±2.72defgh |
| H18 | 5±0.40bc | 281.98±13.42ghijk | 55.38±0.82bc | 42.14±0.73e | 44.62±0.82j | 28.74±1.89jk |
| H19 | 4±0.26cdefgh | 302.93±18.59fghi | 33.68±0.41l | 44.67±0.50cde | 66.32±0.41b | 44.13±0.04ab |
| H20 | 5±0.30bcdef | 231.15±13.58jk | 28.51±0.39m | 56.65±0.97a | 71.49±0.39a | 36.27±0.35defgh |
| H21 | 4±0.40fghij | 220.44±12.32k | 38.52±1.47hijk | 36.96±1.43f | 61.48±1.47def | 38.81±1.19cdef |
| H22 | 3±0.17kl | 465.10±18.66ab | 37.77±0.44hijkl | 49.70±0.74b | 62.23±0.44def | 40.33±0.52bcd |
| H23 | 2±0.25l | 298.04±19.53fghij | 45.97±1.21e | 33.20±1.87g | 54.03±1.21h | 34.28±3.31fghi |
| H24 | 4±0.23ghijk | 336.30±12.50defghi | 35.61±0.57jkl | 46.40±1.19bcd | 64.39±0.57bcd | 45.84±0.04a |
| H25 | 4±0.31defghi | 294.88±14.71fghij | 38.96±0.65ghij | 43.17±0.73de | 61.04±0.65def | 43.39±1.35abc |
| CV (%) | 43.72 | 37.30 | 30.47 | 16.85 | 15.57 | 15.95 |

Table S5. Eigenvalue of each principal component.

| Comprehensive index | PC1 | PC2 | PC3 | PC4 | PC5 |
| --- | --- | --- | --- | --- | --- |
| Fruit weight | 0.35 | 0.13 | 0.02 | -0.13 | 0.19 |
| Fruit transverse diameter | 0.35 | 0.14 | 0.06 | -0.08 | 0.12 |
| Pericarp weight | 0.34 | 0.07 | 0.00 | -0.29 | 0.13 |
| Yield per square metre of canopy | 0.14 | 0.41 | -0.14 | 0.18 | -0.08 |
| Land equivalent oil yield | 0.14 | 0.41 | -0.14 | 0.18 | -0.08 |
| Seed number per plant | 0.16 | 0.33 | 0.23 | 0.00 | -0.09 |
| Plant height | 0.07 | -0.14 | 0.45 | 0.00 | 0.30 |
| Dry seed rate | -0.20 | 0.21 | 0.43 | -0.15 | 0.17 |
| Seed oil content | -0.22 | 0.16 | 0.33 | -0.15 | 0.08 |
| Fresh seed rate | -0.10 | 0.17 | 0.12 | 0.51 | 0.32 |
| Yield per plant | 0.28 | 0.19 | 0.04 | 0.29 | -0.17 |
| Pericarp thickness | 0.24 | -0.07 | -0.03 | -0.49 | 0.03 |
| Fruit longitudinal diameter | 0.28 | -0.03 | -0.13 | -0.05 | 0.46 |
| 100-seed weight | 0.14 | -0.12 | -0.17 | 0.26 | 0.48 |

Table S6 Mean value of trains of five groups.

| Terms | Traits | Group I | Group II | Group III | Group IV | Group V |
| --- | --- | --- | --- | --- | --- | --- |
| Tree architecture | Plant height (m) | 2.28 | 2.47 | 2.93 | 2.40 | 2.39 |
|  | Crown width (east-west) (m) | 2.06 | 2.33 | 2.30 | 2.13 | 2.18 |
|  | Crown width (north-south) (m) | 2.24 | 2.39 | 2.64 | 2.13 | 1.88 |
|  | Canopy area (m^2^) | 3.72 | 4.49 | 4.91 | 3.86 | 3.31 |
| Fruit yield | Yield per plant (kg) | 1.24 | 1.05 | 0.64 | 2.16 | 3.19 |
|  | Yield per square meter of canopy (kg/m^2^) | 0.32 | 0.24 | 0.13 | 0.53 | 1.12 |
|  | land equivalent oil yield (kg/ha) | 247.92 | 184.92 | 97.68 | 413.67 | 876.41 |
| Fruit quality | Fruit weight (g) | 28.41 | 29.77 | 36.18 | 32.39 | 37.21 |
|  | Pericarp weight (g) | 16.15 | 16.71 | 22.19 | 17.30 | 20.94 |
|  | Pericarp thickness (mm) | 4.01 | 4.30 | 4.72 | 3.87 | 4.49 |
|  | Fruit longitudinal diameter (mm) | 37.53 | 38.79 | 40.59 | 38.51 | 39.49 |
|  | Fruit transverse diameter (mm) | 37.33 | 37.67 | 40.69 | 39.50 | 41.62 |
| Seed and oil | 100-seed weight (g) | 312.58 | 354.79 | 388.50 | 327.35 | 296.36 |
|  | Seed moisture content (%) | 42.23 | 50.14 | 35.96 | 38.39 | 43.61 |
|  | Fresh seed rate (%) | 42.04 | 42.91 | 40.78 | 47.00 | 43.36 |
|  | Dry seed rate (%) | 57.77 | 54.96 | 64.08 | 61.62 | 56.39 |
|  | Seed oil content (%) | 34.28 | 34.85 | 39.24 | 37.13 | 34.27 |
